# *Palmaria palmata *(Dulse) as an unusual maritime aetiology of hyperkalemia in a patient with chronic renal failure: a case report

**DOI:** 10.1186/1752-1947-4-301

**Published:** 2010-09-08

**Authors:** Brent M McGrath, John Paul Harmon, Graham Bishop

**Affiliations:** 1 Department of Medicine (General Internal Medicine), Dalhousie University, and Saint John Regional Hospital, Department of Medical Education, 3DS, 400 University Avenue, PO Box 2100, Saint John, NB, E2L 4L2, Canada; 2 Department of Medicine (Respirology), Saint John Regional Hospital, 400 University Avenue, Saint John, NB, E2L 4L2, Canada

## Abstract

**Introduction:**

Hyperkalemia is rare in individuals with normal renal function, regardless of dietary intake. This is due to the ability of the kidneys to adapt to increasing serum potassium concentrations. In patients with renal compromise, potassium homeostasis can become impaired. *Palmaria palmata *(dulse) is an edible seaweed known to be very rich in potassium. We report a case of hyperkalemia precipitated by the consumption of dulse by a patient with known renal disease.

**Case Presentation:**

A 66-year-old Caucasian woman with diabetes and chronic renal disease presented to our emergency department with nausea, vomiting, and worsening malaise, which had been present for less than a day. She had undergone electrocardiogram monitoring, which showed bradycardia, and periods of asystole. Our patient denied any other symptoms. Laboratory analysis revealed a serum potassium level of 8.6 mmol/L (normal range 3.5 to 4.9 mmol/L). Although our patient was taking some medications known to influence renal function, the only recent change that she could recount was that she had consumed approximately 200 g of dulse within the preceding 24 hours. A diagnosis of hyperkalemia was made, and the patient was treated successfully, and discharged home in her pre-morbid state.

**Conclusion:**

To the best of our knowledge, this is the first published report of hyperkalemia due to dulse consumption. Dulse is high in potassium, with concentrations upwards of 34 times greater than that found in bananas. Caution should be taken in prescribing medications with potential adverse renal effects for patients with known renal impairment. In such instances, renal function should be monitored closely. Patients should be counseled to avoid dietary sources high in potassium, with particular attention paid to unusual geographical dietary variations.

## Introduction

Hyperkalemia is rare in individuals with normal renal function, regardless of dietary intake. This is due to the ability of the kidneys to adapt to increasing serum potassium concentrations. In patients with renal impairment, potassium homeostasis can become impaired. *Palmaria palmata *(dulse) is an edible seaweed known to be very rich in potassium. We describe a case of hyperkalemia precipitated by the consumption of dulse in a patient with known renal disease.

## Case Presentation

**Figure 1:**
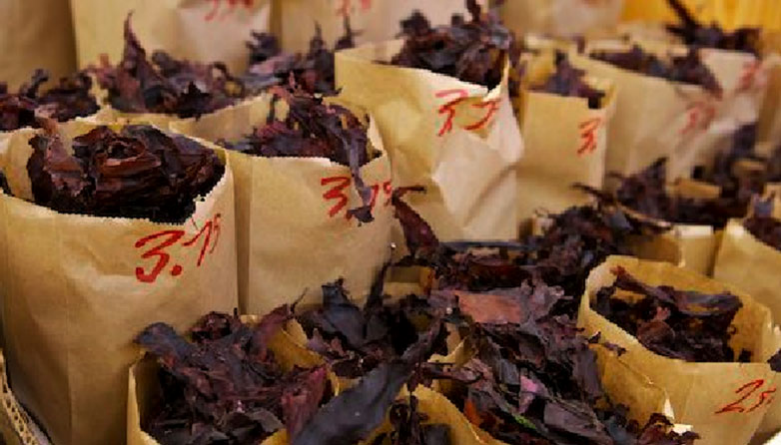
**Dried seaweed (dulse) consumed as a snack food**. Dulse is packaged in brown paper bags (28 cm high by 13.5 cm wide by 9 cm deep) that have a volume of 3400 cm^3^, and are sold based on weight.

A 66-year-old, non-smoking Caucasian woman was transferred to our emergency department after she had presented to a community health clinic with nausea, vomiting, and worsening malaise that had been present for less than a day. An electrocardiogram (EKG) performed at the clinic showed bradycardia, and periods of asystole. Our patient denied any other symptoms, including chest pain, shortness of breath, and syncope. The only recent change that she could recount was that she had consumed approximately 200 g of dulse the previous day (Figure 1).

Our patient's medical history included type 2 diabetes mellitus, hypertension, hypercholesterolemia, gout, anxiety, and chronic renal disease. Her medications on presentation included omeprazole, allopurinol, lorazepam, rosuvastatin, ezetimibe, losartan, metformin, pioglitazone, amlodipine, acebutolol, famciclovir, nortriptyline, hydroxyzine, and hydrocortisone acetate cream. None of the medications were new to the patient.

On physical examination, we found our patient to be an alert, and oriented, obese woman of stated age, with a Glasgow coma scale score of 15. Her heart rate was irregularly irregular, and bradycardic at 30 beats per minute, blood pressure was 124/82 mmHg, and respirations were tachypnic at 28 breaths per minute, with an oxygen saturation of 98% on 100% oxygen administered by facemask. The remainder of the physical examination was unremarkable.

**Figure 2:**
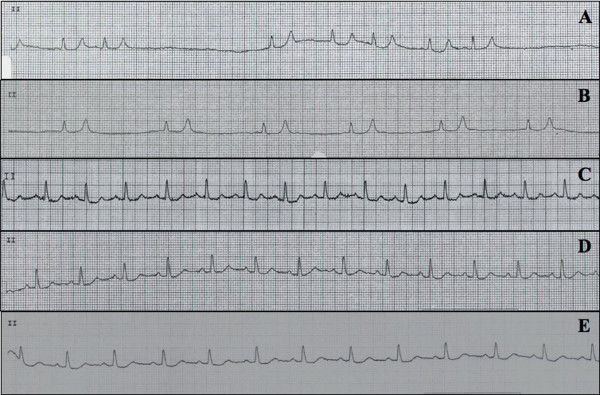
**(A-E) EKG changes from initial presentation, and after treatment initiation**. EKG tracing from lead II at (A) initial presentation, (B) about one hour after initial presentation, (C) about three hours after initial presentation, (D) about 13 hours after initial presentation, and (E) at discharge.

Laboratory investigation revealed an elevated white blood cell count, normal hemoglobin, normal platelets, and a random glucose level of 10.2 mmol/L (reference range 3.0 to 6.0 mmol/L). Testing for cardiac enzymes was negative. Arterial blood gas revealed a compensated respiratory alkalosis, with a pH of 7.40, PaCO_2 _of 33 mmHg, PaO_2 _of 80 mmHg, and a HCO_3 _of 21 mEq/L. Renal function was impaired, with elevated serum urea (11.9 mmol/L; normal range 2.5 to 7.5 mmol/L), and creatinine (168 μmol/L; 60 to 110 μmol/L). Estimation of the patient's glomerular filtration rate revealed a creatinine clearance (CrCl) rate of 37 ml/min (normal range 88 to 128 ml/min), which placed her chronic kidney disease at stage 3b by the National Kidney Foundation criteria [1]. Our patient was also found to be severely hyperkalemic, with a serum potassium concentration of 8.6 mmol/L (normal range 3.5 to 4.9 mmol/L). Her EKG rhythm on presentation to the emergency department showed an irregularly irregular bradycardic rhythm with periods of asystole, absent P waves, widened QRS complex, and peaked t waves (Figure 2).

We diagnosed our patient with hyperkalemia secondary to acute on chronic renal failure, precipitated by the consumption of a large amount of potassium-rich dulse. She was admitted to the intensive care unit, and started on a treatment regimen that included insulin, bicarbonate, calcium gluconate, and sodium polystyrene sulfonate (Kayexalate - Sanofi-Aventis, Laval, Quebec, Canada). Our patient's usual doses of allopurinol, metformin, and losartan were withdrawn, and ultimately discontinued.

Our patient showed marked improvement overnight, and was discharged to the ward. Within a few hours of treatment initiation, her serum potassium had almost completely normalized (5.5 mmol/L), as did the EKG changes (Figure 2A, and 2B). By the next morning, she was eukalemic (4.1 mmol/L), with a normal sinus rhythm (Figure 2C). Her serum creatinine level also improved, and at discharge was close to the patient's pre-admission baseline (93 μmol/L), with an estimated CrCl of 66 ml/min, indicating stage 2 chronic kidney disease [1]. She remained stable with a normal potassium, and cardiac rhythm, and was discharged on hospital day seven (Figure 2D).

## Discussion

Dulse, the colloquial name given to *Palmaria palmata*, is a common red seaweed, inhabiting the rocky marine coastline, and tidepools [2]. It is named because of its resemblance to the palmar surface of the hand (Latin, *palma*). The use of dulse can be dated back to the 12th century in Ireland. Although it can be consumed directly after harvesting, it is most often served sun-dried. In the Maritime Provinces of eastern Canada, dulse is widely available, and consumed as a snack food [3]. However, there are few available data on its consumption patterns. Dulse is known to be high in potassium, with reported concentrations ranging from 22.2 to 122 mg/g of dry weight [4]. By comparison, bananas (*Musa acuminata colla*), a well-known dietary source of potassium, have a reported potassium content of around 3.58 mg/g of edible portion [5,6]. To put this into perspective, the 200 g of dulse consumed by the patient is equivalent (in terms of weight) to approximately two peeled bananas, but contains upwards of 34 times the concentration of potassium. The average daily dietary intake of potassium is between 1550 and 4700 mg, over 90% of which is absorbed via the gastrointestinal tract [7].

Hyperkalemia is seldom seen in well individuals with normal renal function, regardless of dietary intake. The vast majority (90%) of the daily intake of potassium is excreted in urine, with the remaining 10% excreted in stool. This makes renal function essential for effective potassium management. Hyperkalemia can result from any one or a combination of all three mechanisms: (1) a shift in potassium concentration from the intracellular environment to the extracellular fluid, (2) an increase in dietary potassium intake, and/or (3) impairment in renal potassium excretion [7]. It is our contention that although this patient was predisposed to developing hyperkalemia by her illness, and medications, its precipitation was triggered by the high potassium load obtained from the dulse. In this way, her illness, and medications were necessary for the hyperkalemia, but not in, and of themselves causative.

Treatment of hyperkalemia involves correcting the underlying pathology, protecting cardiac tissue from the toxic effects of potassium, shifting potassium from the extracellular to the intracellular compartment, and ultimately increasing potassium excretion [8-11].

## Conclusion

To the best of our knowledge, this is the first published report of hyperkalemia secondary to dulse consumption. Patients with known renal impairment should be cautiously prescribed medications with potential adverse renal effects. In such instances where such medications are necessary, renal function should be monitored closely. Moreover, patients should be counseled to avoid dietary sources high in potassium, with particular attention paid to unusual geographical dietary variations.

## Competing interests

The authors declare that they have no competing interests.

## Consent

Written informed consent was obtained from the patient for publication of this case report and accompanying images. A copy of the written consent is available for review by the Editor-in-Chief of this journal.

## Authors' contributions

BMM, and JPH contributed to the conception, and writing of this case report. BMM performed the search of the literature. GB provided expert review of earlier drafts, and was the lead physician involved in the intensive care management of the patient. All authors have read, and approved the final manuscript.
